# COMPARISON OF HEPATIC PROFILE IN PRE AND POSTOPERATIVE OF BARIATRIC SURGERY: PRIVATE VS PUBLIC NETWORK

**DOI:** 10.1590/S0102-6720201500040014

**Published:** 2015

**Authors:** Taianne Machado NASCIMENTO, Antônio ALVES-JÚNIOR, Marco Antonio Prado NUNES, Tiago Rodrigo Pereira de FREITAS, Marco Antonio Fontes Sarmento da SILVA, Maria Rosa Melo ALVES

**Affiliations:** University Hospital, Federal University of Sergipe, Aracaju, SE, Brazil.

**Keywords:** Non-alcoholic Fatty Liver Disease, Hepatic fibrosis, Obesity, Bariatric Surgery

## Abstract

**Background:**

Obesity is associated to several comorbidities, including nonalcoholic fatty liver disease, which implicates in isolated steatosis to steatohepatitis. The latter may progress to severe manifestations such as liver fibrosis, cirrhosis and hepatocellular carcinoma.

**Aim:**

To compare the presence of advanced liver fibrosis before and after bariatric surgery in patients of private and public health system.

**Methods:**

Patients from public and privative networks were studied before and after bariatric surgery. The presence or absence of advanced hepatic fibrosis was evaluated by NAFLD Fibrosis Score, a non-invasive method that uses age, BMI, AST/ALT ratio, albumin, platelet count and the presence or absence of hyperglycemia or diabetes. The characteristics of the two groups were compared. The established statistical significance criterion was p<0.05.

**Results:**

Were analyzed 40 patients with a mean age of 34.6±9.5 years for private network and 40.6± 10.2 years for public. The study sample, 35% were treated at private health system and 65% in the public ones, 38% male and 62% female. Preoperatively in the private network one (7.1%) patient had advanced liver fibrosis and developed to the absence of liver fibrosis after surgery. In the public eight (30.8%) patients had advanced liver fibrosis preoperatively, and at one year after the proportion fell to six (23%).

**Conclusion:**

The non-alcoholic fatty liver disease in its advanced form is more prevalent in obese patients treated in the public network than in the treated at the private network and bariatric surgery may be important therapeutic option in both populations.

## Abstract (pt)

**Racional:**

A obesidade está relacionada com diversas comorbidades, entre elas a doença hepática gordurosa não-alcoólica, que inclui desde esteatose isolada à esteatohepatite. Esta última pode evoluir para manifestações mais graves, como fibrose hepática, cirrose e hepatocarcinoma.

**Objetivo:**

Comparar a presença de fibrose hepática avançada antes e após cirurgia bariátrica em pacientes da rede particular e pública.

**Métodos:**

Foram estudados pacientes antes e após operação bariátrica das redes particular e pública. A presença ou ausência de fibrose hepática avançada foi avaliada através do NAFLD Fibrosis Score, método não invasivo que utiliza idade, IMC, relação AST/ALT, albumina, contagem de plaquetas e presença ou ausência de hiperglicemia ou diabete. As características dos dois grupos foram comparadas O critério de significância estatística estabelecido foi p<0,05.

**Resultados:**

Foram analisados 40 pacientes com idade média de 34,6±9,5 anos para rede particular e 40,6±10,2 anos para pública. Da amostra avaliada, 35% foram atendidos no sistema de saúde particular e 65% no sistema público, sendo 38% gênero masculino e 62% feminino. No pré-operatório da rede particular um (7,1%) paciente apresentou fibrose hepática avançada e evoluiu para ausência de fibrose hepática após o procedimento cirúrgico. Já na rede pública oito (30,8%) apresentaram fibrose hepática avançada no pré-operatório, sendo que no pós de um ano esse valor foi para seis (23%).

**Conclusão:**

A hepatopatia gordurosa não alcóolica na forma avançada é mais prevalente em pacientes obesos atendidos na rede pública do que nos da rede privada, sendo que a cirurgia bariátrica pode ser importante opção terapêutica em ambas populações.

## INTRODUCTION

The increase of body weight is a significant public health challenge^11^. It is estimated that more than 1.9 billion adults are overweight, and 600 million of these are obese^24^. In the United States, over 16.9% of young people and 34.9% of adults are considered obese^16^. In Brazil the prevalence of overweight in adults living in the capitals of the 26 states and the Federal District increased from 43.2% in 2006 to 51.0% by 2012^13^. If these recent trends continue, in 2030 to 57.8% of the adult population (3.3 billion people) will be overweight or obese^11^.

Obesity is a risk factor for several diseases, contributing to the global load of incapacitating and chronic diseases^15^. Pathological conditions associated with obesity include some of the following disorders: cardiovascular, endocrine, respiratory, gastrointestinal, skin, genitourinary, musculoskeletal, neoplasms, psychosocial and some other implications, such as increased anesthetic and surgical risk and decreased physique alertness^23^. Obesity today is linked to more worldwide deaths than underweight^24^.

Clinical treatment is the first option. It usually includes the use of anorectic or disabsorptive medicines, besides psychological treatments, physical therapy, dietary and exercise^5^. However, clinical treatment does not deliver long-term significant results, while bariatric operation is the most effective tool to control and treat morbid obesity. Although invasive, it has achieved satisfactory results, leading to the reduction of more than 50% of the overweight or 30 to 40% of the initial weight. It aims the reduction of hunger signals and increased satiety, thus, producing a controlled state of undernutrition^10^^,^ 17 ^,^ 19.

Nonalcoholic fatty liver disease (NAFLD) is a condition defined by the excessive accumulation of fat that is not related to alcoholic consumption. This accumulation had its prevalence doubled during the past 20 years and it occurs in the form of triglycerides (steatosis), exceeding 5 to 10% of liver weight. A subset of patients presenting hepatic steatosis will evolve with steatohepatitis, dramatically increasing the risk of cirrhosis, liver failure and hepatocellular carcinoma. Currently, NAFLD and nonalcoholic steatohepatitis are considered the number one cause of hepatic illnesses in Western countries^8^^,^ 12 ^,^ 21.

Abdominal ultrasound is the most frequently used complementary exam to diagnose hepatic steatosis both in clinical evaluations and in epidemiological studies. Despite the lower accuracy compared to tomography and magnetic resonance imaging and not being able to distinguish steatosis from liver steatohepatitis, this procedure has to be the easiest, due to its noninvasive method. Additionally, it presents as less expensive when compared to other image methods^4^^,^ 7.

Liver biopsy is considered the gold standard for directly diagnosing NAFLD and evaluating the inflammation/fibrosis; however, its use is limited because it is invasive, has a high cost and presents sampling error or inadequate sample quantity. Thus, several noninvasive methods using panels of markers or counts, instead of biopsy, to identify patients with steatohepatitis or fibrosis, are being proposed. However, suitable decision algorithms validated for clinical practice are still scarce^14^.

A non-invasive test example is the NAFLD Fibrosis Score, developed and validated by Angulo et al. (2007). This scale measures the degree of advanced liver fibrosis from the calculated score based on six variables: age, BMI, relative AST/ALT, albumin, platelet count and presence or absence of hyperglycemia or diabetes to identify or exclude advanced liver fibrosis, defined as stages 3 and 4 of the proposed classification by Brunt, which evaluates the fibrotic stage of the histology based on five points^1^^,^ 18.

The aim of this study was to compare the presence of nonalcoholic fatty liver disease in obese patients before and after bariatric surgery between the private and public network.

## METHOD

Patients participated in this study by their own free will, and have undergone the signature of a term of consent. The research project was approved by the Ethics Committee of Human Research at the Federal University of Sergipe, under Protocol No 17402613.1.0000.5546.

The initial sample was 65 patients from the public network and 107 patients from the private network after bariatric surgery by the same surgeon and accompanied by the same multidisciplinary team, therefore, subjected to even pre, intra and postoperatively accompanying protocol. It was excluded those who did not agree to participate, did not have sufficient postoperative time, did not follow up on services or whose necessary data to complete the study were not available, patients with no evidence of hepatic steatosis by imaging and other suspected causes of liver disease, as assessed by clinical examination and serology for hepatitis B, hepatitis C and HIV. Alcohol consumption was evaluated through interviews with patients, here defined as exclusion factor a weekly consumption higher than 210 g for men and 140 g for women^1^. After applying the exclusion criteria, the final study sample was composed of 40 patients, among them 14 from the public network and 26 from the private. Patients of the public network have been seen, operated and monitored in the University Hospital of the Federal University of Sergipe. The private network patients have been seen in private offices and operated in private hospitals. In both cases, they had the same surgical team, same nutritional and psychological protocols.

The patients underwent vertical banded gastroplasty with gastrojejunal derivation in Roux-en-Y. Anthropometric characteristics were evaluated (gender, age, weight, BMI) and laboratory (blood glucose, platelet counts and determination of serum aspartate aminotransferase - AST, alanine aminotransferase - ALT and albumin) and the presence or absence of hyperglycemia in the preoperative and postoperative period of six months and one year.

The presence or absence of advanced hepatic fibrosis was assessed by the Non-Alcoholic Fatty Liver Disease Fibrosis Score (NAFLD Fibrosis Score). For the calculation of the index it was used: age in years, BMI in kg/m², determination of serum AST and ALT U/l, serum albumin in g/dl, and the presence of diabetes mellitus and hyperglycemia (blood glucose equal or higher than 110 mg/dl). The calculation was performed according to the following formula: NAFLD Fibrosis Score = -1.675 + 0.037 × age (years) + 0.094 × BMI (kg/m²) + 1.13 × hyperglycemia/diabetes mellitus (yes=1, no=0) + 0.99 × AST / ALT ratio - 0.013 × platelets (× 10⁹/l - 0.66 x Albumin, g/dl). Values below -1.455 indicate absence of fibrosis advanced liver and above 0.676 the presence of advanced hepatic fibrosis. Values from -1.455 to 0.676 are considered indeterminate regarding the presence of advanced fibrosis.

Data analysis was performed using descriptive statistics in which the categorical variables were expressed as absolute and relative frequencies and numeric variables were presented as central tendency measurements and variability. The characteristics of the two groups were compared by the chi-square test in the case of categorical variables and the Student's t-test for independent samples in the case of numerical variables. For repeated measures, analysis of variance (ANOVA) was used for comparisons between groups and moments (preoperatively, six months and one year). The established statistical significance criterion was p<0.05.

## RESULTS

Forty patients were analyzed, of which 35% (14/40) were seen at supplemental health system and 65% (26/40) in the public health system. The entire sample had a mean age of 39 years (CI95%: 35-42 years); the vast majority, 62%, of females (27/40) and only 38% male (13/40). From the private network, 11 were women (79%) and three men (21%) and from the public network 16 women (62%) and 10 men (38%). (Table 1)

**TABLE 1:** - Clinical data characterization of preoperative patients treated at private and public network

	**Private**	**Public**	**Total**	**p**		
	**n**	**%**	**n**	**%**		
Gender						
Male	3	21%	10	38%	13	0,316
Female	11	79%	16	62%	27	
Hyperglycemia - diabetes mellitus						
No	11	79%	17	65%	28	0,484
Yes	3	21%	9	35%	12	
Fibrosis degree						
No fibrosis	10	71%	6	23%	16	0,011
Indeterminate	3	21%	12	46%	15	
With fibrosis	1	7%	8	31%	9	
Total	14	100%	26	100%	40	

The average BMI in the preoperative private and public networks were respectively 39.1±4.7 kg/m² and 47.8± 2.3 kg/m²; after postoperative six months follow-up these values were 29.5±4.9 kg/m² and 35.6±8.7 kg/m²; and in the postoperative period of one year values were 26.9±4.0 kg/m² and 32.8±9 kg/m², observing statistically significant reduction (p<0.001).

In relation to the private network, there was a significant statistical difference when collated preoperative and postoperative individual values of BMI and weight (p<0.05); however, AST, ALT, albumin and NAFLD did not represent statistical differences (p> 0.05). Table 2 shows the characterization of the private network patients concerning anthropometric and laboratory variables evaluated.

**TABLE 2:** - Characteristics of the private network sample during periods of observation: preoperative, six months and 12 months of postoperative.

	**Preoperative Average (SD)**	**Six-month Average (SD)**	**One-year Average (SD)**	**Total Average (SD)**	**p**
BMI	39,1 (4,7)	29,5 (4,9)	26,9 (4,0)	31,8 (7,0)	< 0,001
AST	27,2 (12,0)	25,1 (9,0)	32,1 (31,4)	28,2 (19,8)	0,649
ALT	37,5 (33,3)	27,8 (17,1)	31,4 (16,8)	32,2 (23,4)	0,477
Albumin	4,1 (0,26)	4,0 (0,33)	4,2 (0,39)	4,1 (0,33)	0,341
Weight	108,1 (24,4)	81,6 (21,7)	73,7 (13,0)	87,8 (24,8)	< 0,001
NAFLD	-2,1212 (1,65)	-2,5088 (1,19)	-3,0400 (0,93)	-2,5567 (1,32)	0,086

BMI= Body Mass Index; NAFLD score= nonalcoholic fatty liver disease

As for the public, there was significant statistical difference when compared preoperative and postoperative individual values of BMI, weight and NAFLD. AST, ALT and albumin values did not show statistical differences. Table 3 shows the characterization of public network patients concerning anthropometric and laboratory variables evaluated.

**TABLE 3:** - Characteristics of the public network sample during the following periods of observation: preoperative, six months and 12 months of postoperative.

	**Preoperative Average (SD)**	**Six-month Average (SD)**	**One-year Average (SD)**	**Total Average(SD)**	**p**
BMI	47,8 (12,3)	35,6 (8,7)	32,8 (9,0)	38,8 (12,0)	< 0,001
AST	25,2 (9,5)	30,0 (16,2)	29,8 (19,7)	28,3 (15,7)	0,296
ALT	35,9 (25,6)	36,7 (19,2)	33,8 (17,6)	35,5 (21,0)	0,788
Albumin	3,8 (0,48)	3,8 (0,52)	3,9 (0,54)	3,9 (0,51)	0,663
Weight	128,5 (34,3)	95,9 (24,7)	87,9 (22,6)	104,1 (32,6)	< 0,001
NAFLD	-0,6845 (2,17)	-1,3298 (1,68)	-1,6898 (1,91)	-1,2347 (1,96)	0,0002

BMI=Body Mass Index; NAFLD score=nonalcoholic fatty liver disease.

By applying the NAFLD Fibrosis Score, before the operation, the presence of preoperative advanced liver fibrosis was identified in one (7.1%) patient in the private network and eight (30.8%) in public; the intermediate degree of fibrosis and absence of fibrosis were for private and public networks of, respectively, three (21.4%) and 12 (46.2%); and 10 (71.4%) and six (23.1%) in this period, presenting statistical significance (p <0.05) (Table 1).

**FIGURE 1:**
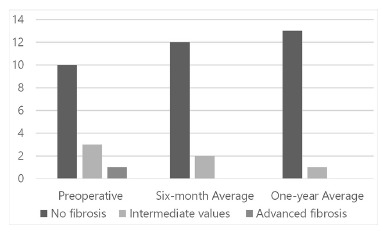
- Graphic representation of the number of patients served by supplementary care network, with the degree of fibrosis liver defined by NAFLD (nonalcoholic fatty liver disease)

After the surgery, the presence of advanced liver fibrosis was not identified in any patient of the private network, in both of the six-month and one-year postoperative. This group obtained intermediate values and without fibrosis in the postoperative period of six months, respectively, two (14.3%) and 12 (85.7%), and after 12 months, one (7.1%) and 13 (92.9% - Figure 1)

**FIGURE 2:**
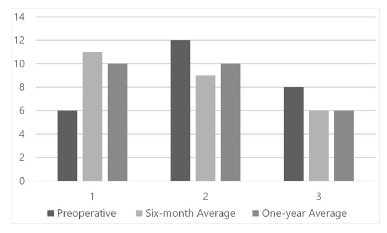
- Graphic representation of the number of patients served by public health, with the degree of liver fibrosis defined by NAFLD (nonalcoholic fatty liver disease)

In public network, six (23.1%) patients had advanced fibrosis during the period after six months and one year. In this network, there were intermediate values of fibrosis: nine (34.7%) in the postoperative period of six months and 10 (38.5%) in a year. It was classified as no fibrosis 11 (42.3%) patients after six months and 10 (38.5%) in a year of the public network (Figure 2).

From the sample evaluated, among the nine patients with advanced fibrosis before operation, one (11.1%) stopped presenting advanced fibrosis and three (33.3%) went to the indeterminate range. Regarding the 15 patients with indeterminate score, seven (46.7%) stopped presenting postoperative advanced fibrosis, whereas seven (46.7%) remained with indeterminate score and one (6.7%) evolved with indeterminate fibrosis. The three patients without preoperative advanced fibrosis remained so after the surgery.

Except for two (5%) patients, all have reduced the value of score after the procedure.

## DISCUSSION

The prevalence of obesity is increasing^24^, which contributes to the load of global chronic diseases^15^. One of these diseases is NAFLD, having obesity as one of the main risk factors^3^. The justification for this phenomenon is that the increase of the release of free fatty acids, resistin, IL-6 and TNF-alpha by the adipose tissue and the reduction of the release of adiponectin contribute to the development of insulin resistance in obesity and increased risk of developing NAFLD^6^.

Other studies determine the prevalence of non-alcoholic liver disease in patients referred for bariatric surgery. Marceau et al. demonstrated prevalence of 86% to steatosis, 23% to steatohepatitis and 2% to cirrhosis among 551 patients^9^.

In the present study, the mean BMI before operation was higher for public patients 47.8± 2.3 kg/m², which rates it, in level of obesity, greater than the private network 39.1±4.7 kg/m². These data corroborate the literature, since there is an inverse relationship between the socioeconomic level and the prevalence of obesity. Furthermore, there is also an association between income and consumption of leafy vegetables; thus, individuals with lower incomes are less likely to consume these foods, which are important for low-calorie diet and healthy eating habits^20^.

In the overall sample, 27 (67.5%) were women and 13 (32.5%) men. Among the private network, there were 11 women (79%) and three (21%) men and the public network was composed of 16 (62%) women and 10 (38%) men. Another aspect observed in the studies is the predominance of women in the cases of bariatric surgery in Brazil. This may be related to the higher prevalence of obesity among females, to the different determinants of obesity between the sexes and/or to the fact that obese women may show greater motivation for the pursuit of weight loss, mainly due to social pressures^2^.

There were differences in terms of age (p=0.078) showing values of 34.6±9.5 years for private and 40.6±10.2 years for public networks. This can be a consequence of the average waiting time difference of the patients for concluding the transaction by SUS and by the Supplementary Health Network. In SUS, patients wait for 2.9 years, while patients who have private health insurance can carry it out with the minimum time interval since the first appointment, only having to meet the clinical requirements for realizing it^22^.

The prevalence of steatohepatitis not alcoholic in the US population is of 30%, while in patients undergoing bariatric surgery is 90%. The identification of the presence and severity of hepatic fibrosis in patients with steatosis liver is crucial for the subsequent orientation management, since the ones with fibrosis have higher risk for cirrhosis, portal hypertension, hepatocellular carcinoma and death^18^.

Among the 40 patients, nine had preoperative advanced liver fibrosis; of these, eight (88.9%) were from the public network, responsible for a higher mean BMI than the private one. This is in agreement with the literature, because according to the National Health and Nutrition Examination Survey III, the prevalence of primary non-alcoholic liver disease increases according to the BMI^9^.

Of the 14 patients in the private network, 13 (92.8%) had a <-1.455 score in the postoperative of one year and, thus, the absence of advanced liver fibrosis. Only one patient in the preoperative was classified as a carrier of the disease, which evolved after the weight loss to the fibrosis resolution. In the public network, there were 26 patients and after a year procedure, the number of advanced fibrosis patients was six (23%). The number of those who did not have fibrosis preoperatively rose from six to 10 (38.4%) after one year, demonstrating the therapeutic effect of bariatric surgery and weight loss caused by it.

The private network evolved with a higher percentage of patients without advanced liver fibrosis after a year of surgery; this probably is closely related to the lower BMI of these patients when compared to the public network ones.

Both obesity and nonalcoholic fatty liver disease are rising diseases and deserve further studies and papers involving their relations. Including the continuation of this research to remedy some limitations, such as the expansion of the casuistry and the follow-up time to ratify the results found herein.

## CONCLUSION

Non-alcoholic fatty liver disease in an advanced form is more prevalent in obese patients seen in the public network than those in private ones, and bariatric surgery may be an important therapeutic option in both populations.
